# Revenge Fantasies Expressed Through Drawings and Narratives: Insights from Indian Perspectives Based on Gender and Religion

**DOI:** 10.1007/s11013-025-09928-1

**Published:** 2025-07-18

**Authors:** Meghna Girish, Rachel Lev-Wiesel

**Affiliations:** 1 Graduate School of Creative Arts Therapies, University of Haifa, Haifa, Israel; 2 MA Expressive Arts Therapy Programme, Chulalongkorn University, Bangkok, Thailand; 3 The FAA–Emili Sagol Creative Arts Research and Innovation for Well-Being Center, Chulalongkorn University, Bangkok, Thailand; 4 The Emili Sagol Research Center for Creative Arts Therapies, The Sagol Lab for Children at Risk, University of Haifa, Haifa, Israel; 5 Head of the Body & Mind Psychotherapy track, Social Work, Tel Hai Academic Center, Kiryat Shmona, Israel; 6 Head of the National Center for Children at Risk Assessment, The Sagol Center for Hyperbaric Treatment and Research, Shamir Hospital, Be’er Ya’akov, Israel

**Keywords:** Revenge fantasies, Indians, Religious differences, Gender, Drawings, Art-based assessment

## Abstract

The mixed-methods study aimed to explore revenge fantasies among Indians, focusing on gender and religious differences, and to evaluate the alignment between quantitative measures and qualitative expressions through drawings and narratives. The sample comprised 97 Indian women and 55 men, aged 18–56, who identified as either Hindu or Christian. Quantitative assessments included the demographics sheet, Traumatic Events Questionnaire (TEQ), and Injustice Experiences Questionnaire (IEQ). Qualitative measures involved drawings and narratives depicting a personal injustice and the participant's desired outcome for the perpetrator. Analysis employed non-parametric tests and Interpretative Phenomenological Analysis for the drawings and narratives. The findings revealed no overall gender differences in the revenge fantasies depicted in drawings, though differences emerged in the types of perpetrators and central themes in narratives. Religious affiliation influenced the type of revenge fantasy, with Hindus and Christians showing significant differences in narrative organization, central themes, and resolution. Additionally, significant correlations were found between IEQ scores and various drawing indicators (event type, perpetrator type, and hierarchy) as well as narrative themes. These results suggest that gender and religious affiliation intricately shape revenge fantasies, highlighting the importance of considering cultural and social factors in understanding responses to perceived injustices.

## Introduction

Revenge is a psychological phenomenon that emerges in response to perceived injustice or trauma, often manifesting as anger or embitterment (Elshout et al., 2017). This reaction, frequently described as the “urge to fight back,” occurs across a variety of situations, including interpersonal conflicts, bullying, workplace disputes, and broader experiences of trauma (McKee & Feather, 2008). Intrusive and persistent revenge fantasies often accompany these emotions, driven by feelings of anger toward the perpetrator (Horowitz, 2007). Engaging in such fantasies allows individuals to cope by ruminating on ways to regain control and a sense of justice. This desire for revenge serves as both a coping mechanism and a way to alleviate feelings of vulnerability (Sievers & Mersky, 2006). However, despite the temporary empowerment that revenge fantasies provide, they also risk perpetuating anger and rumination (Anderson & Bushman, 2002; Haen & Weber, 2009). The presence of revenge fantasies and the desire for revenge has been observed across societies, with individuals from various cultural backgrounds experiencing them as part of human cognition and emotional processing (Bloom, 2001; McCullough, 2008).

Despite the pervasiveness of revenge fantasies globally, limited research has examined them in the socio-cultural contexts within India. Cultural norms influence emotional expression across societies, including in India, where social expectations may shape how emotions related to socially sensitive topics, such as revenge, are articulated. Research has shown that ruminating on anger and feelings of injustice can predict poor physical health and heightened stress among Indian youth (Suchday, 2015; Suchday et al., 2007). Given the emphasis on social interconnectedness in many Indian communities, individuals may suppress their revenge-related thoughts to maintain social harmony (Nagai, 2007). This highlights the importance of exploring revenge fantasies within Indian cultural and religious contexts, as social and cultural factors, including norms around emotional expression and social expectations, can shape how individuals report or experience thoughts related to revenge.

While several studies have investigated the role of personality traits and emotional responses in revenge fantasies (Barcaccia et al., 2022; Elshout et al., 2017; Rasmussen, 2016; Stoia-Caraballo et al., 2008), there remain notable gaps in the literature, particularly regarding gender and religious influences on revenge in India. In a country with a highly diverse population and deeply ingrained religious beliefs, these factors are crucial in shaping how individuals respond to perceived injustices. Most research on revenge fantasies has relied on quantitative questionnaires, which are prone to social desirability biases. This limitation calls for a mixed-methods approach that combines quantitative and qualitative methods to gain a more nuanced understanding of revenge fantasies in such settings. The use of drawings and narratives, alongside surveys, allows for the exploration of implicit beliefs and emotions that may not be easily captured through direct questioning.

Revenge fantasies, defined as cognitive processes where individuals mentally devise ways to retaliate against those who have wronged them, often become intrusive and emotionally charged (Horowitz, 2007; Yoshimura & Boon, 2023). These fantasies serve a psychological function, providing temporary relief and a sense of empowerment by helping individuals feel in control of a situation where they otherwise feel powerless (Sievers & Mersky, 2006). While such fantasies may alleviate feelings of vulnerability in the short term, they can also lead to further rumination, increasing emotional distress (Knafo, 2004). Engaging in revenge fantasies can also evoke intense emotions such as shame, guilt, and hatred toward the perpetrator, fueling continued cycles of anger and resentment (Haen & Weber, 2009; Seebauer et al., 2014). Despite these psychological costs, revenge fantasies are thought to offer a means of self-protection, particularly when individuals anticipate further injustice.

The role of gender in shaping revenge fantasies is an important area of study, as societal norms on gender can influence how individuals express aggression. Gender role socialization encourages men to exhibit aggression, while women are often socialized to prioritize empathy and relational harmony; much of the existing literature on gender and aggression being conducted in Western or generalized contexts (Björkqvist, 2018; Gault & Sabini, 2000). In India, aggression and retaliation are shaped by complex cultural factors, including notions of honor, shame, and gender roles evidenced in studies among Indian adolescents that revealed significant gender differences in anger regulation; males exhibiting higher physical aggression and females more relational hostility (Phogat & Singh, 2021; Sharma et al., 2017; Sidhu et al., 2019). These patterns underscore the importance of considering gender when studying revenge fantasies in India. Research specific to Indian populations suggests that gendered expressions of aggression and revenge may vary considerably across different cultural contexts. For instance, studies such as those by (Girish & Lev-Wiesel, 2024) highlight the nuanced ways in which Indian Hindu women navigate expressions of anger and revenge in response to injustice. Given the diversity within India, it is essential to consider how gender socialization may differ across communities, shaping revenge fantasies.

Religious beliefs and teachings on justice and retribution can influence how individuals conceptualize and express revenge fantasies. In India, Hinduism is the dominant religion, practiced by around 80% of the population, while Christianity is a minority faith (Census Tables | Government of India, n.d.; Kramer, 2021). Hinduism, with its emphasis on karma and dharma, promotes a balance between peace and conditional acceptance of violence (King, 2022). Religious texts such as the Ramayana and Mahabharata advocate for non-violence and cosmic justice, yet they also contain narratives that can reinforce revenge and retaliation in response to injustice. This duality is particularly evident in the experiences of Hindu women, who may face patriarchal pressures that contribute to revenge fantasies (Penna, 1989). Christianity, by contrast, emphasizes forgiveness and divine justice, with teachings from the New Testament (Matthew 5:38-39) encouraging believers to “turn the other cheek” rather than seek revenge (Carlsmith et al., 2008). Examining how individuals from Hindu and Christian backgrounds conceptualize revenge offers valuable insights into how religious teachings shape responses to injustice. While research on this topic is limited, Lev-Wiesel and colleagues (2023a) research on the Jewish population provides a relevant comparison, showing how religious identity influences revenge-related thoughts.

Autobiographical memory of traumatic events often leads to fragmented narratives (Midgley, 2003). Drawing is a therapeutic tool that allows individuals to express their emotions and experiences (Lev-Wiesel et al., 2020; Malchiodi, 2014). It can express hidden distress acceptably, helping communicate difficult emotions (Cobia & Brazelton, 1994; Peterson & Hardin, 1997). Healing requires creating a coherent narrative of trauma (Brewster & Zimmerman, 2022), and it is seen that drawings with narratives are effective in processing and expressing traumatic experiences (Lev-Wiesel & Liraz, 2007). A study on child sexual abuse victims found that these tools helped them express feelings of isolation and desire for retaliation against their abusers (Lev-Wiesel et al., 2023b). Feelings of insult may sometimes be downplayed in certain social contexts to maintain harmony, a dynamic that can be observed across various cultures (Nagai, 2007). Religious beliefs, while playing an important role in shaping societal values, do so in complex ways that are not unique to any one country, and such influences are seen in various global contexts (Edara, 2017). Using indirect methods like drawings and narratives can uncover implicit beliefs about revenge, often seen as morally wrong (Kaufman, 2013) and difficult to assess with direct means such as questionnaires. Employing a mixed-methods research design hence could be beneficial when researching revenge fantasies.

Given the importance of gender, religious beliefs, and cultural norms in shaping revenge fantasies, the present study aims to investigate these factors among Indian adults using a mixed-methods approach. By integrating qualitative methods, such as drawings and narratives, with quantitative measures from questionnaires, this research seeks to provide a comprehensive understanding of perspectives within the Indian socio-cultural landscape on revenge fantasies. Specifically, the study addresses the following research questions: (1) Do revenge fantasies in drawings and narratives of Indian adults differ by gender? (2) Do they differ between Christians and Hindus? (3) Is there an association between the revenge fantasies assessed through qualitative (drawings and narratives) and quantitative measures?

## Materials and Methods

### Participants and Procedure

The study used a mixed methods design, incorporating both quantitative and qualitative measures to explore revenge fantasies. After receiving ethical approval from the University of Haifa (146/19), 152 participants (97 women, 55 men), aged 18–56 (*M* = 26.33, *SD* = 8.45) residing in India, were recruited via convenience sampling. Invitations to participate in the study were circulated over social media and social networks. Inclusion criteria required participants to be 18 years or older, be fluent in English with Native language belonging to one of the major Dravidian languages (i.e. Tamil, Telugu, Tulu, Kannada, Malayalam) and have no clinical diagnosis of major psychological disorders. Participants from all religious backgrounds, including Muslims, were initially welcome to participate in the study. However, only a small percentage of Muslim participants gave their consent during data collection, which left the sample size too small for a meaningful analysis. Consequently, only Hindu and Christian participants—who made up the majority of the sample—were included in the final analysis for the purposes of this study. Those who completed the measures and matched the inclusion criteria were included in the sample. Almost 86% spoke Malayalam and remaining (14%) were non-Malayalam speakers. 71% belonged to early adulthood (18–29 years) and remaining (29%) fell into the middle age range (30-56 years). The religious affiliation distribution included 91 Hindus and 61 Christians. Most participants were single (77.63%), held at least a Bachelor’s degree (38.81%), and reported verbal or emotional abuse (54.60%) as their most common traumatic event. Table 1 has the distribution of the demographic variables. Participants were asked to create two drawings on A4 white paper, with reassurance that the drawings didn’t need to be perfect or aesthetically pleasing. The first drawing depicted a personal unjust event and the second illustrated their revenge fantasy. After completing the drawings, participants wrote narratives for each and filled the questionnaires. Confidentiality and anonymity were assured, and participants had the right to withdraw at any time. Ethical guidelines were followed, with informed consent obtained and contact information for counselling services provided if needed after the study.

**Table 1:** Distribution of demographic variables for the entire sample (N = 152)

Demographic variables	Mean [SD]/N (%)
Language	
Malayalam	131 (86.2)
Telugu	7 (4.6)
Tamil	11 (7.2)
Kannada/Tulu	3 (1.9)
Age	26.38 (8.45)
Age category	
Early adulthood (18–29 years)	108 (71.1)
Middle aged (30–56 years)	44 (28.9)
Gender	
Females	97 (63.8)
Males	55 (36.2)
Religious affiliation	
Hindus	91 (59.9)
Christians	61 (40.1)
Marital status	
Single	118 (77.6)
Married	26 (17.1)
Not together	8 (5.3)
Education level	
High school education	37 (24.3)
Bachelor’s	59 (38.8)
Master’s	49 (32.2)
MPhil / PhD	7 (4.6)

### Measures

#### Qualitative Measures

Participants created two sequential drawings in response to the prompts: “Draw an unjust event you experienced” and “Draw what you would prefer to happen to the person who unjustly treated you.” The drawings, completed with pencil or pen on A4 paper, were accompanied by written narratives explaining each drawing. Both the drawings and narratives were analyzed using the Interpretative Phenomenological Analysis (IPA) to explore the participants’ subjective experiences of revenge fantasies. Given IPA’s focus on meaning-making, particular attention was paid to how individuals constructed and expressed their revenge fantasies through visual and verbal representations. In order to organize the data, neutral descriptions of the drawings and narratives was documented. This was followed by analysis of spatial and symbolic elements such as size, position, barriers, connections and symbols. Visual cues were connected with the verbal descriptions and themes were coded from both drawings and narratives. Shared psychological experiences were identified by looking for patterns across participants. Visual themes and written narratives were linked for a holistic understanding which gave an integrated interpretation.

A previously developed coding system (Lev-Wiesel et al., 2023a) was used, with relevant codes adapted to fit this study’s cultural and thematic focus. The qualitative analysis was conducted by experienced art therapists experienced in both clinical and research contexts. Additionally, new codes specific to Indian culture were created, and themes were inductively generated to address the research questions. To ensure rigor, inter-rater reliability was assessed for content and style indicators, with only those meeting the substantial agreement range (0.61—0.80) included in the final analysis. Concordance between revenge fantasies in drawings and narratives was also evaluated, deepening the interpretative understanding of how participants construct revenge-related meaning across modalities. To structure the analysis, content and style indicators (key components in artwork analysis (Gantt, 2004; Matto & Naglieri, 2005) and art perception (Augustin et al., 2011)) informed the interpretative process while maintaining the idiographic nature of IPA. Content indicators for the drawings included the type of unjust event, perpetrator type, hierarchy of the perpetrator (whether perpetrators were peers, family members, employers, teachers or colleagues, children), specificity of the perpetrator, and presence of multiple perpetrators or not. Drawing style indicators assessed whether the self was depicted, drawing type, size of the victim, and size of the perpetrator. Narratives were coded for organization, dissociation, central theme, and resolution, with attention to how revenge fantasies were articulated verbally versus visually.

#### Quantitative Measures

##### Demographic Sheet

The information collected was the participant’s age, religious affiliation, marital status, and educational level.

##### The Traumatic Events Questionnaire (TEQ)

A checklist of 9 specific types of traumatic events from the Traumatic Events Questionnaire (Vrana & Lauterbach, 1994), namely car accident, physical abuse, terror attack or war, hospitalization due to illness, sexual abuse, loss of family member, social exclusion, verbal or emotional abuse, and other events. Participants could mark more than one event and each event was coded as one if it occurred and zero if it never happened. The number of events reported was summed to determine the severity of the trauma history.

##### The Injustice Experiences Questionnaire (IEQ)

The IEQ is a 12-item questionnaire assessing the sense of injustice in post-injury life (Sullivan, 2008), particularly cognitions associated with unfairness, perceived severity, the irreparability of loss, and blame. Some of the items are “I cannot believe this happened to me”, “I feel that this has affected me in a permanent way”, and “It all seems so unfair”. Responses are on a 5-point Likert scale, ranging from 0 (never) to 4 (all the time), with a strong reliability of 0.92. There are two subscales, namely Blame (6 items) and Severity (6 items). In the current study, a Cronbach’s alpha of 0.89 was seen, and the scores did not follow normal distribution. The median and Interquartile range for IEQ Total scores were 21 and [12; 29] respectively.

### Data Analysis

Data was not normally distributed as assessed using the Shapiro–Wilk test (*p* > 0.05), hence non-parametric tests were used. Mean and standard deviation reported continuous variables, while frequencies and percentages reported categorical variables. The Chi-square test or Spearman’s correlation assessed associations between categorical or continuous variables. Wilcoxon rank-sum and Kruskal Wallis tests were used to find differences in continuous scores over categorical variables. For the qualitative analysis, Interpretative Phenomenological Analysis (IPA) was applied to both drawings and narratives to explore how participants made sense of unjust experiences and their revenge fantasies. The multimodal approach outlined by Boden et al. (2019) was used to integrate visual and textual expressions, allowing for a richer, layered understanding of participants’ perspectives. Narratives were further analyzed following Josselson and Lieblich’s (2003) framework, emphasizing the subjective and emotionally embedded nature of revenge fantasies. Analysis was done using R Version 4.3.3. Statistical significance was at *p*-value < 0.05.

## Results

### Qualitative Findings

The qualitative analysis of drawings and narratives revealed five overarching themes in how participants expressed their revenge fantasies: (1) revenge by proxy, (2) passive aggression, (3) direct action, (4) forgiveness, and (5) avoidance. These themes emerged through participants’ lived experiences and were reflected in their artistic and written expressions.

i. *Revenge by Proxy.* Participants who expressed revenge by proxy often depicted external forces—divine justice, societal retaliation, or legal consequences—as the means through which perpetrators would be punished. Drawings commonly featured symbols of higher authority, such as judges, police officers, or supernatural elements like a deity with halo, surrounded by clouds. A female participant’s (Hindu) drawing showed a deity towering over a small figure i.e. the participant, with figures under her showing reconciliation with the perpetrator. The accompanying narrative stated: *“Drawing 2 depicts that time changes everything and here I let God take care of it. God is watching everything and everyone no bad wish on anyone if something meant to be will always come to you”.* Another male participant (Christian) drew a table with 3 chairs set up for supper. The narrative accompanied is as follows: *“The truth is, my God turned this around for good. I would like to see those involved at the table of my heavenly Father—feasting at His table, enjoying His food as I enjoyed the food during my stay there.”.* The belief in external justice mechanisms appeared to provide psychological distance from personal acts of vengeance.
ii. *Passive Aggression.* Expressions of passive aggression included fantasies where perpetrators experienced humiliation, social exclusion, or psychological distress rather than physical harm. Drawings often depicted perpetrators in scenarios and symbols such as a depiction of the Christian cross, alongside scenes of public shame, unemployment, or relationship failures. A male participant (Hindu) described his drawing of a former friend: *“Most people don’t realise the value of having someone who can listen and clear their emotional burdens. Their presence is often undervalued and taken for granted. Their absence somehow will be when all the noise settles down and when they really feel alone. I just want them to recognise this and take it as a learning experience and try to find happiness from within”.* Another female participant (Christian) described her drawing: “*I really don’t know. I don’t want them to suffer but I do want them to suffer. I want to pray for their mental peace and will hope they learn their lesson.”* Her drawing depicted a cross and a small sized figure.
iii. *Direct Action.* The most overt expressions of revenge involved direct harm to the perpetrator. Participants in this category depicted violent retribution, ranging from physical assault to extreme forms of punishment such as symbols of knife, acid bottle, objects to attack with. One female participant’s (Hindu) narrative was: “*The person who mistreated me said that I will throw acid to you. He threatened me by saying so I would like to do the same.”.* A male participant (Christian) depicted direct action in his drawings with symbols showing that he would throw an object (Pepsi can) back at the perpetrator. This was his narrative: “*An Arab guy in Dubai was rude after he saw a Pepsi can near me and told me that this is not India because he thought I would throw it on the street after finishing the drink”.* The narratives within this category often conveyed a sense of moral justification for the act, suggesting a cognitive framing that legitimized the aggression.
iv. *Forgiveness.* A set of participants expressed a desire to forgive rather than seek revenge. Their drawings often included reconciliatory gestures, such as two figures shaking hands, embracing, or simply moving forward in separate directions. One female participant (Christian) described their decision to let go of resentment: *“Listening and speaking between the two of us. Clarifying the problem and forgiving”.* A male participant (Hindu) narrated: *“I drew my dad to be happier than before. Have more occasions to smile. If he finds joy around others, I want him to experience the joy more frequently”.* Forgiveness was sometimes framed as an active decision rather than an absence of anger, with participants emphasizing personal growth and resilience.
v. *Avoidance.* Avoidance emerged as a distinct theme in which participants distanced themselves from the perpetrator physically, emotionally, or mentally. Drawings in this category frequently depicted blank spaces, missing figures, or even no reference of the injustice. One female participant’s (Hindu) drawing featured themselves with a check mark above, when previously she had a X mark above, with narrative: *“I wish things were positively favourable for me too. Like getting acknowledged by Sir and selecting me for the school team.”.* A male participant (Christian)’s narrative was as follows: “*I hope we get actual gender equality. Society should treat everyone in a same way in everything”.* For these participants, revenge was not a central focus; instead, their narratives suggested an emphasis on self-preservation and healing.

### Quantitative Findings

**Fig. 1:**
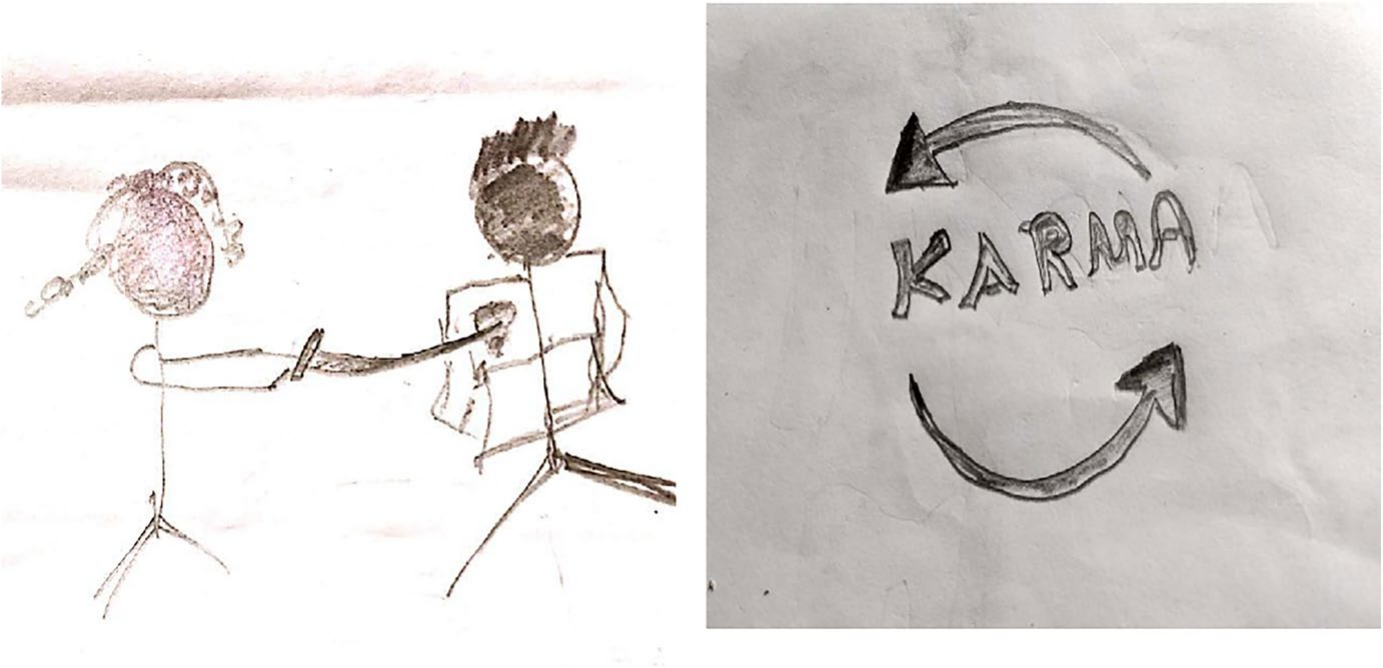
29 aged Hindu male’s unjust event and revenge fantasy drawing with Verbal or emotional abuse listed in TEQ

**Fig. 2:**
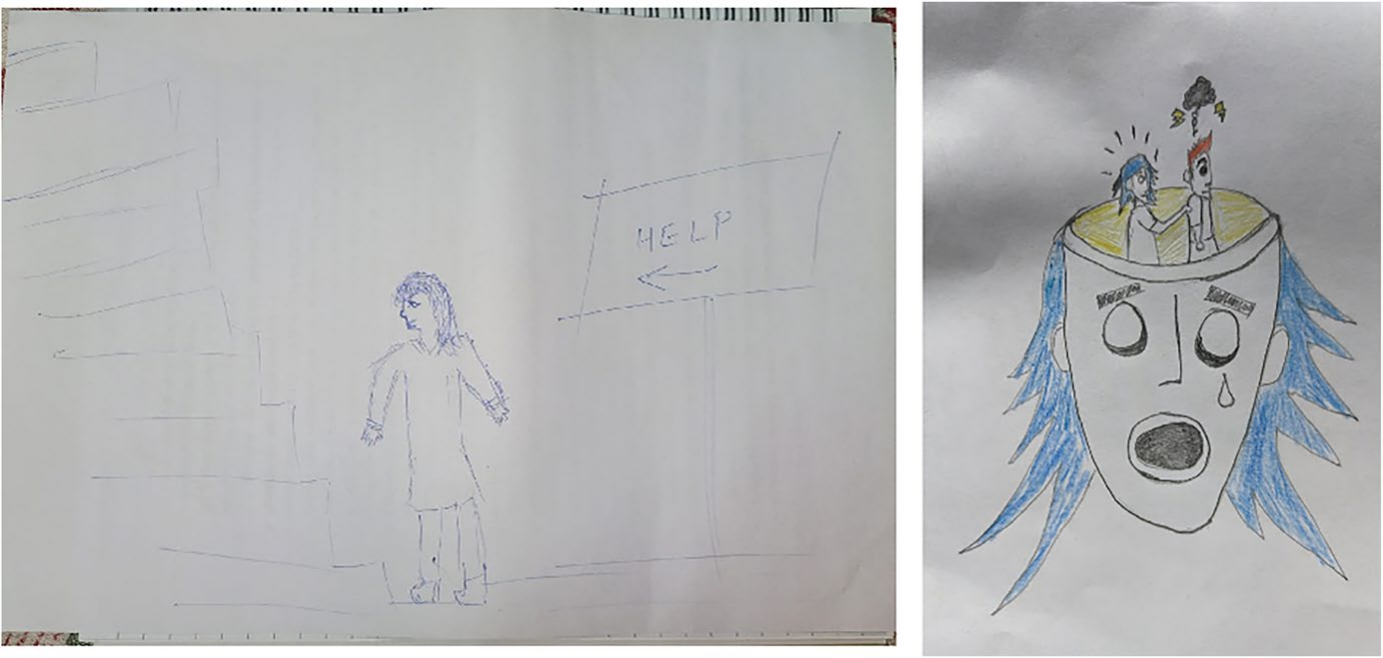
Comparison of revenge fantasy of 21 aged female versus 34 aged male (both Hindus) as avoidance and passive aggression respectively, with non-family and peers as perpetrators

**Fig. 3:**
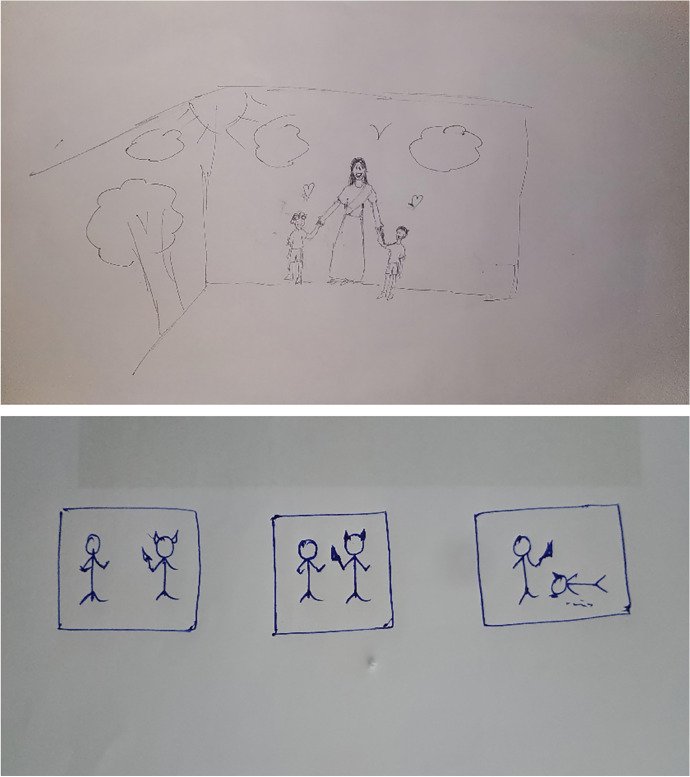
Comparison of revenge fantasy of 21 aged female (Christian) versus 22 aged male (Hindu) as avoidance and direct action respectively, with non-family and peers as perpetrators

The following tables show the quantitative analysis (see Table 2, 3, 4, 5, 6, 7). Examples of drawings from participants can be found in  Figures 1, 2, and 3).

**Table 2:** Comparison of drawing style related indicators between unjust event drawing and revenge fantasy drawing (N = 152)

Variables	Unjust event (Drawing 1)	Revenge fantasy (Drawing 2)	χ2	*p*-value	Effect size
	n (%)	n (%)			
Self-depicted or not			10.47	0.0012*	0.27
Yes	121 (79.6)	81 (53.3)			
No	31 (20.4)	71 (46.7)			
Drawing type			142.49	0.000**	0.68
Figurative style	65 (42.8)	60 (39.5)			
Stick figure style	70 (46.1)	63 (41.4)			
Metaphoric or abstract	17 (11.2)	29 (19.1)			
Size of the victim			28.85	0.000**	0.25
Tiny	60 (39.5)	28 (18.4)			
Normative	63 (41.4)	51 (33.6)			
Exaggerated	4 (2.6)	8 (5.30)			
Omitted	25 (16.4)	65 (42.8)			
Size of the perpetrator			46.92	0.000**	0.32
Tiny	45 (29.6)	26 (17.1)			
Normative	67 (44.1)	73 (48.0)			
Exaggerated	12 (7.9)	13 (8.6)			
Omitted	28 (18.4)	40 (26.3)			

*p < 0.01. **p < 0.001.

**Table 3:** Gender differences in revenge fantasy in drawings and narratives

Variables	Gender		χ2	*p*-value
	Females	Males		
	n (%)	n (%)		
Revenge fantasy from drawings			6.18	0.186
By proxy	16(10.52)	9 (5.92)		
Passive aggression	8 (5.26)	7 (4.60)		
Direct action	16 (10.52)	6 (3.94)		
Forgiveness	8 (5.26)	11 (7.23)		
Avoidance	49 (32.23)	22 (14.47)		
Revenge fantasy from narratives			4.72	0.316
By proxy	17 (11.18)	6 (3.94)		
Passive aggression	28 (18.42)	20 (13.15)		
Direct action	10 (6.57)	7 (4.60)		
Forgiveness	12 (7.89)	11 (7.23)		
Avoidance	30 (19.73)	11 (7.23)

**Table 4:** Gender differences in revenge fantasy related indicators in drawings and narratives

Variables	Gender		χ2	*p*-value	Effect size
	Females	Males			
	n (%)	n (%)			
From drawings					
Unjust event			0.44	0.8003	
Domestic	24 (15.78)	11 (7.23)			
External	53 (34.86)	32 (21.05)			
Unspecified	20 (13.15)	12 (7.89)			
Perpetrator type			19.5	0.00061*	0.34
Authority figures and society	5 (3.28)	17 (11.18)			
Family and close relations	22 (14.47)	8 (5.26)			
Known others (non-family)	22 (14.47)	7 (4.60)			
Peers and associations	19 (12.5)	9 (5.92)			
Stranger or unspecified	29 (19.07)	14 (9.21)			
Specified vs unspecified			1.23	0.268	
Yes	58 (38.15)	27 (17.76)			
No	39 (25.65)	28 (18.42)			
Multiple perpetrators			0.05	0.814	
Yes	16 (10.52)	11 (7.23)			
No	78 (51.31)	44 (28.94)			
Hierarchy of the perpetrator			0.21	0.9002	
Lower/same level	26 (17.10)	13 (8.55)			
Upper	48 (31.57)	29 (19.07)			
Unspecified	23 (15.13)	13 (8.55)			
From narratives					
Narrative organization			5.76	0.055	
Coherent—short	67 (44.07)	31 (20.39)			
Coherent—long	20 (13.15)	12 (7.89)			
Incoherent	8 (5.26)	12 (7.89)			
Narrative dissociation			2.68	0.101	
Yes	34 (22.36)	28 (18.42)			
No	61 (40.13)	27 (17.76)			
Central theme in narrative			18.2	0.002*	0.35
Aggression and conflict	8 (5.26)	8 (5.26)			
Authority and system related injustice	6 (3.94)	14 (9.21)			
Body and gender related issues	25 (16.44)	5 (3.28)			
Emotional and relational issues	26 (17.10)	11 (7.23)			
Forgiveness and letting go	5 (3.28)	5 (3.28)			
Trauma and victimization	27 (17.76)	12 (7.89)			
Resolution in the narrative			0.43	0.806	
Positive	33 (21.71)	19 (12.5)			
Negative	23 (15.13)	11 (7.23)			
Neutral	29 (19.07)	25 (16.44)			

*p < 0.001.

**Table 5:** Religious affiliation differences in Revenge fantasy in drawings and narratives

Variables	Religion		χ2	*p*-value	Effect size
	Hindus	Christians			
	n (%)	n (%)			
Revenge fantasy from drawings			9.535	0.04*	0.25
By proxy	18 (11.84)	7 (4.60)			
Passive aggression	13 (8.55)	2 (1.31)			
Direct action	11 (7.23)	11 (7.23)			
Forgiveness	8 (5.26)	11 (7.23)			
Avoidance	41 (26.97)	30 (19.73)			
Revenge fantasy from narratives			2.32	0.676	
By proxy	16 (10.52)	7 (4.60)			
Passive aggression	29 (19.07)	19 (12.5)			
Direct action	10 (6.57)	7 (4.60)			
Forgiveness	11 (7.23)	12 (7.89)			
Avoidance	25 (16.44)	16 (10.52)			

*p < 0.05.

**Table 6:** Religious affiliation differences in revenge fantasy related indicators

Variables	Religion		χ2	*p*-value	Effect size
	Hindus	Christians			
	n (%)	n (%)			
From drawings					
Unjust event			3.851	0.145	
Domestic	24 (15.78)	11 (7.23)			
External	45 (29.60)	40 (26.31)			
Unspecified	22 (14.47)	10 (6.57)			
Perpetrator type			19.52	3.435	
Authority figures and society	12 (7.89)	10 (6.57)			
Family and close relations	21 (13.81)	9 (5.92)			
Known others (non-family)	16 (10.52)	13 (8.55)			
Peers and associations	14 (9.21)	14 (9.21)			
Stranger or unspecified	28 (18.42)	15 (9.86)			
Specified vs unspecified			2.139	0.143	
Yes	46 (30.26)	39 (25.65)			
No	45 (29.60)	22 (14.47)			
Multiple perpetrators			0.268	0.604	
Yes	18 (11.84)	9 (5.92)			
No	72 (47.36)	50 (32.89)			
Hierarchy of the perpetrator			0.951	0.621	
Lower/Same level	22 (14.47)	17 (11.18)			
Upper	45 (29.60)	32 (21.05)			
Unspecified	24 (15.78)	12 (7.89)			
From narratives					
Narrative organization			11.78	0.0027**	0.28
Coherent—short	64 (42.10)	34 (22.36)			
Coherent—long	11 (7.23)	21 (13.81)			
Incoherent	15 (9.86)	5 (3.28)			
Narrative dissociation			0.056	0.812	
Yes	36 (23.68)	26 (17.10)			
No	54 (35.52)	34 (22.36)			
Central theme in narrative			11.708	0.039*	0.278
Aggression and conflict	13 (8.55)	3 (1.97)			
Authority and system related injustice	12 (7.89)	8 (5.26)			
Body and gender related issues	15 (9.86)	15 (9.86)			
Emotional and relational issues	23 (15.13)	14 (9.21)			
Forgiveness and letting go	2 (1.31)	8 (5.26)			
Trauma and victimization	26 (17.10)	13 (8.55)			
Resolution in the narrative			6.5092	0.0386*	0.208
Positive	24 (15.78)	28 (18.42)			
Negative	22(14.47)	12 (7.89)			
Neutral	44 (28.94)	20 (13.15)			

*p < 0.05. **p < 0.01.

**Table 7:** Correlation between IEQ and drawing/narrative indicators

Variables	Blame	Severity	IEQ Total
Specified/vague perpetrator	0.175*	0.07	0.136
Multiple perpetrators or not	− 0.14	− 0.243**	− 0.204*
Narrative dissociation	− 0.12	− 0.12	− 0.12

*p < 0.05. **p < 0.01.

A Wilcoxon rank-sum test was conducted to compare the IEQ scores of females and males (*N* = 152). The results did not show a significant difference in scores between gender (W = 2827, *p* = 0.54). However, when IEQ scores were compared between religious affiliations, there was a significant difference between Hindus (Median = 23) and Christians (Median =18), W = 3351, *p* = 0.03 (see Table 8).

**Table 8:** Kruskal Wallis test results on IEQ with drawing and narrative indicators

Variables	N	Mean rank	χ2	df	*p*-value	
Revenge fantasy from drawing			3.25	4	0.515	
By proxy	25	89.5				
Passive aggressive	15	68.33				
Direct action	22	71.65				
Forgiveness	19	71.05				
Avoidance	71	76.6				
Revenge fantasy from narrative			5.09	4	0.278	
By proxy	23	91.06				
Passive aggressive	49	69.09				
Direct action	18	87.08				
Forgiveness	24	77.39				
Avoidance	42	79.54				
Unjust event type			13.885	2	0.0009***	
Domestic	35	95.18				
External	85	76.85				
Both/unspecified	32	55.1				
Perpetrator			14.39	4	0.0061**	
Authority figures and society	22	65.1136				
Family and close relations	30	94.6833				
Known others (non-family)	29	81.4655				
Peers and associations	28	86.1964				
Stranger/unspecified	43	59.9767				
Hierarchy of the perpetrator			6.613	2	0.036*	
Lower/same level	39	85.487				
Upper level	77	79.35				
Unspecified	36	60.66				
Narrative organization			4.0088	2	0.134	
Coherent short	98					
Coherent long	32					
Inherent	20					
Central theme in narrative			12.85	5	0.024*	
Aggression and conflict	16	100.84				
Authority and system related injustice	20	66.75				
Body and gender related issues	30	72.3				
Emotional and relational issues	37	80.68				
Forgiveness and letting go	10	41.95				
Trauma and victimization	39	79.62				
Resolution in the narrative			1.796	2	0.407	
Positive	52	83.49				
Negative	34	71.36				
Neutral	64	75.39				

*p < 0.05. **p < 0.01. ***p < 0.001.

Post-hoc pairwise analysis revealed that except for Unjust event type, there were no significant differences. Those participants whose drawings showed domestic events reported higher IEQ scores than those whose drawings showed unspecified events (*p* < 0.01).

### Integrating Qualitative and Quantitative Findings

Both the qualitative and quantitative findings reveal meaningful connections between revenge fantasy types and individual differences in gender and religion (Christian versus Hindus). There were no significant associations between the revenge fantasy types and the IEQ for the entire sample (*p* > 0.05). The unjust event type had significant differences in IEQ scores.

A series of tests were run between demographic variables and revenge fantasies measured through the IEQ. IEQ scores did not vary with language, age category (early adulthood and middle aged), gender, marital status, education status. But significant difference was seen in the IEQ scores (*p =* 0.03; Cohen’s d = 0.35) for religious affiliation i.e. Christians (Mean rank = 67.06) and Hindus (Mean rank = 82.83). While IEQ scores and revenge fantasy types did not vary with gender, variations emerged in the types of perpetrators depicted and the themes of the narratives. Men tended to focus more on authority figures and strangers, with their narratives emphasizing systemic injustices and oppression by power structures. Women, on the other hand, depicted more strangers and known figures, with narratives highlighting personal trauma and relational injustices.

When analysing religious affiliation, there were no significant differences between drawing related indicators but only in narrative related indicators. Religious affiliation was significantly associated with narrative organization with Hindus having short, coherent narratives than Christians. Narratives by Hindus had greater percentage of trauma and victimization in comparison to Christians. Finally, resolution in the narrative differed significantly by religious affiliation. Christians had a slightly higher percentage of positive resolutions, while Hindus had more neutral resolutions outcomes.

## Discussion

The study aimed to explore revenge fantasies expressed through qualitative and quantitative means to examine the influence of gender and religious affiliation (Christians and Hindus) among Indian adults. Analysis of the two types of drawings revealed significant differences in the presence or omission of self, drawing style, and the sizes of both the victim and perpetrator across the sample. Gender differences in revenge fantasies were noted only in the type of perpetrator and the central theme of the narrative. However, religious affiliation was associated with differences in the revenge fantasy type in drawings and other narrative related indicators. Significant correlations were found between the specification of the perpetrator or the presence of multiple perpetrators and scores on the Injustice Experiences Questionnaire (IEQ). While IEQ scores did not vary by type of revenge fantasy in drawings or narratives, there were notable differences in the IEQ scores based on type of unjust event; domestic events scoring highest on IEQ in comparison to external or unspecified events. IEQ scores also differed between Christians and Hindus.

A comparison between the drawings of unjust events and revenge fantasies revealed that participants were less likely to depict themselves (omission of self) in the revenge fantasy drawing, and preferred abstract or metaphorical styles instead. This could be indicative of a shift in emotional or cognitive processing, with participants moving from literal to symbolic representations from the unjust event drawing to the revenge fantasy drawing. The finding suggests a tendency toward abstraction, omission, and depersonalization in revenge fantasies when individuals imagine retaliatory scenarios, in line with psychoanalytic lens (Horowitz, 2007). The size of the victim changed between the two drawings. In the revenge fantasy drawing, there was a notable increase in omitted victims, indicating a possible psychological distancing from the victim role revenge fantasies. The size of the perpetrator also changed with fewer tiny depictions and more omitted or enlarged perpetrators. The possible explanation is maybe the participants expressed greater emotional distance in the revenge fantasy, using it as a coping mechanism to handle the emotional impact of the event. Many participants reported revenge fantasies based on karmic justice, divine vengeance, personal healing, or ethical restraint when they left themselves out of their drawings or downplayed the victim and offender figures. Some of the narrative descriptions of the drawings were “Attachment kills. Karma will take care”, “May they be guided and enlightened by Gandhian values”, “She needs help... should seek help and get rid of insecure negative thoughts”, and “Honestly nothing, I don’t think revenge helps so I don’t want to think about it.” These tendencies might be a reflection of larger cultural frameworks in Indian society, such as collectivist traditions and values that place an emphasis on acceptance, non-violence, and karmic consequences (Verma, 1999). A recent cross-cultural study comparing Indians and Americans found that Indians were less likely to seek direct revenge, tending toward reconciliation or acceptance over retribution (Goyal & Miller, 2023).

No gender differences were observed in revenge fantasies, contrary to previous research (Goldner et al., 2019; Yoshimura & Boon, 2023). It is possible that collective societal values, such as justice, honor, and retaliation, may outweigh gender-specific expectations. In the socio-cultural contexts within India, women might find expressing vengeance through fantasy empowering, especially given the high levels of violence against women, as supported by findings from another study (Girish & Lev-Wiesel, 2024). It is likely that provocation and mistreatment evoke similar feelings of insult and humiliation across genders (Griskevicius et al., 2009). Men often depicted authority figures and strangers, focusing on systemic injustices and perceived oppression from structures like workplaces or legal systems. Experiences of unfair treatment by police officers, educational authorities, employers, and systemic regulations were common. Narratives described incidents such as being unfairly suspended by a principal (“I was suspended by the principal without reason. I still carry that anger. I drew myself symbolically shooting the principal”), being affected by systemic injustices (“A rule changed my life. I was able to overturn it later.”), or facing discriminatory hiring practices (“I wish the company would go bankrupt”). These fantasies suggest a displacement of anger toward broader societal systems rather than direct personal retaliation, which may reflect how patriarchal structures in Indian society (Bhatnagar, 2024) shape masculine experiences of injustice and authority. Rather than confronting personal conflicts, revenge was frequently imagined through the lens of challenging or overturning hierarchical power. Conversely, women depicted more strangers and known figures, indicating concerns about public violence and personal injustices within relationships amidst high levels of emotional abuse and control (Kalokhe et al., 2017; Tripathi et al., 2017). Their narratives centered on trauma and victimization, suggesting that women internalize injustices due to exposure to victimization both within and outside family settings, consistent with a recent study (Girish & Lev-Wiesel, 2024).

A comparison of revenge fantasy types in drawings between Hindus and Christians revealed a significant difference. Hindus were more likely to depict avoidance and indirect forms of retaliation (such as by proxy or passive aggression), while Christians more frequently represented forgiveness. Direct action was equally represented in both groups. These differences may stem from varying cultural and religious beliefs: Hinduism emphasizes karmic consequences, which can involve avoiding the offender or using others as proxy (Goyal & Miller, 2023; King, 2022; Vijayakumar & John, 2018), whereas Christianity places emphasis on forgiveness (VanOyen Witvliet et al., 2008). These findings do not suggest that all Christians in the sample consistently chose forgiveness, nor that Hindus were more inclined toward retaliation. Rather, it raises the question of whether religious teachings might influence how individuals symbolically represent responses to injustice. There was no significant association between revenge fantasy type from narratives and religion. However, Hindus produced shorter, more cohesive narratives, whereas Christians tended to create longer, more elaborate narratives. Christians generally showed a greater predisposition toward forgiveness and positive resolutions in their narratives, while Hindus focused more on personal victimization and conflicts, leading to more negative outcomes. It is possible that it reflects distinct storytelling styles, with Christians potentially reflecting more deeply on the events and Hindus favoring directness and brevity (Evans, 2010). However, there was no significant association between revenge fantasy type in narratives with Christian/Hindu affiliation. It is to be pointed out that higher questionnaire scores were seen among Hindus compared to Christians. The central theme in the narratives also differed significantly with Hindus having greater number of emotional/relational and trauma/victimization themes than Christians. The religious texts in Hinduism, Mahabharata and Ramayana emphasize responsibility, morality, and justice, often set against a backdrop of conflict and the victimization of women. In contrast, the Bible emphasizes forgiveness and redemption, highlighting God’s love for humanity and eternal life through Jesus Christ. Despite the fact that Hindu and Christian participants’ primary narrative themes differed significantly, it is noteworthy that there were more women in the sample among Christians than among Hindus (72% vs. 58%). The gender distribution may have played a role in the observed religious differences, as female participants in our study tended to express more emotional and trauma-related themes. The notable thematic differences, however, are consistent with different emphasis in Christian and Hindu religious teachings, indicating that religious affiliation itself may also independently influence narrative expressions of retaliation and injustice.

Positive correlation with a specified perpetrator for Blame score, and a negative correlation with multiple perpetrators and Severity along with Total IEQ scores were seen. Participants who experienced injustice from multiple perpetrators often minimized their personal sense of injustice, focusing instead on acceptance or personal growth. Several narratives reflected this tendency, with participants expressing sentiments such as “Honestly nothing, I don’t think revenge helps,” “Finding peace within yourself when everyone else has wronged you,” and “I don’t want them to get hurt... let them find peace in whatever they do.” These minimized responses align with Phillips et al.’s (2015) observation that societal discourses in India, particularly around collective acts like gang rape, may discourage individuals from fully externalizing blame when facing harm from multiple perpetrators.Apart from the type of unjust event, there were no significant differences in other drawing or narrative indicators with IEQ scores. Participants whose drawings depicted domestic events reported higher IEQ scores compared to those whose drawings depicted unspecified events. This finding aligns with research showing that the source of injustice significantly impacts perceived severity and blame (Schultz et al., 2010; Watson et al., 2015). Asen and Fonagy (2017) found that family-related cases lead to increased anger and emotional discomfort, a result consistent with this study’s findings.

## Conclusions

### Limitations

The study has several limitations. First, the sample size and uneven distribution across gender and religious groups may limit the generalizability of the findings. The use of a convenient sample also affects representativeness, with a non-normal distribution further compromising results. Additionally, our study had broad religious classifications and did not measure religiosity (frequency of religious practice, adherence to religious teachings) neither the various religious denomination which further limits generalizability. The absence of caste as a primary demographic variable in this study was a significant limitation that limits how the results can be interpreted. Whether expressions of forgiveness reflect religious teachings or the social norms of higher caste status is unclear given the sociocultural nuances in India, especially among Malayali Christian participants who may primarily come from privileged caste backgrounds and comprised as majority of the sample. To better separate these influences, caste information should be included in future studies. These factors could weaken the study’s conclusions, particularly regarding applicability to other populations. Future research should use larger, more diverse samples such as those who have migrated from their hometown to another city, collect religious denominations with caste related information, collect data from other religious affiliations and consider additional variables like socio-economic status, details of the unjust event, and emotional impact to gain deeper insights into revenge fantasies. The influence of language and ethnicity could also be further explored by asking the participants to express narratives of their revenge fantasies in their native language than in English, to explore for nuanced findings specific to ethnic groups.

### Implications

This mixed-methods study is among the first in India to examine how adults express revenge fantasies through drawings and stories, focusing on gender and religious affiliation. While it found no gender-based differences in revenge fantasies, religious affiliation significantly influenced the types of revenge fantasies depicted. By exploring revenge in a non-Western context, the research deepens our understanding of cultural responses to injustice and trauma. The medium to large effect sizes suggests these findings have real therapeutic implications, particularly in art therapy, where shifts in artwork may indicate changes in emotional coping.

## Data Availability

The data supporting this study’s findings are available on request from the corresponding author.
